# A compound score to screen patients with hereditary transthyretin amyloidosis

**DOI:** 10.1007/s00415-022-11056-4

**Published:** 2022-03-13

**Authors:** Stefano Tozza, Daniele Severi, Emanuele Spina, Andrea Di Paolantonio, Aniello Iovino, Valeria Guglielmino, Francesco Aruta, Maria Nolano, Mario Sabatelli, Lucio Santoro, Marco Luigetti, Fiore Manganelli

**Affiliations:** 1 Department of Neurosciences, Reproductive and Odontostomatological Sciences, University of Naples “Federico II”, Via Sergio Pansini, 5, 80131 Naples, Italy; 2 Università Cattolica del Sacro Cuore, Sede Di Roma, Rome, Italy; 3 Fondazione Policlinico Universitario A. Gemelli IRCCS. UOC Neurologia, Rome, Italy; 4 Centro Clinico NEMO Adulti, Rome, Italy

**Keywords:** TTR amyloidosis, Neuropathy, Carpal tunnel syndrome, Neurophysiology, Screening tool

## Abstract

**Background:**

Hereditary transthyretin amyloidosis (ATTRv) is a rare, debilitating and fatal disease, mostly characterized by progressive axonal peripheral neuropathy. Diagnosis is still challenging and diagnostic delay in non-endemic area is about 3–4 years. The aim of this study was to arrange a clinical and electrophysiological score to select patients with axonal neuropathy that deserve screening for TTR mutation.

**Methods:**

Thirty-five ATTRv patients and 55 patients with chronic idiopathic axonal polyneuropathy (CIAP) were retrospectively analyzed. Clinical and electrophysiological findings at first evaluation were collected. Based on significant results between the two groups, a compound (clinical and electrophysiological) score was arranged, and ROC analysis was performed to identify the ideal cut-off able to discriminate between the two groups.

**Results:**

ATTRv patients presented a later age at onset, more frequent muscle weakness and carpal tunnel syndrome history. On the other hand, electrophysiological analysis showed that ATTRv patients had lower CMAP and SAP amplitude in all examined nerves. We arranged a compound score constituted by 7 total items, ranging from 0 to 12. ROC analysis showed an Area Under the Curve = 0.8655 and we set the cut-off ≥ 5 points to discriminate ATTRv patients with a sensitivity of 96.6% and a specificity of 63.6%.

**Conclusion:**

Our study demonstrated that our compound score with cut-off ≥ 5 allows to discriminate ATTRv patients among subject affected by axonal polyneuropathy with a sensitivity > 95%. Thus, our compound score is a quick, easy and effective screening tool.

## Introduction

Hereditary transthyretin-related amyloidosis (ATTRv; v for “variant”) is a multisystem disorder, caused by mutations in *transthyretin* (TTR) gene. It is an autosomal-dominant, debilitating, progressive and fatal disease. Mutated TTR tetramer is unstable, dissociates in misfolded monomers that accumulate in extracellular spaces forming oligomers and amyloid fibrils [1].

Transthyretin-mutated protein accumulates mainly in heart and peripheral nervous system (PNS), leading cardiomyopathy and progressive axonal peripheral neuropathy, respectively [2]. PNS involvement is the presenting complaint in most cases of ATTRv. Polyneuropathy is due to the accumulation of TTR fibrils in PNS causing an axonal length-dependent sensory–motor and autonomic neuropathy [3]. ATTRv is a progressive and invalidating neuropathy and over few years, patients accumulate disability, become wheelchair bound and bedridden, and ultimately die [2].

In the last decade, different treatments (tafamidis, diflunisal, patisiran, inotersen) have proved their efficacy in slowing progression of neuropathy [4–6] and cardiomyopathy [7]. These treatments drastically changed natural history of neuropathy, even though therapies are more efficacious if they are precociously administered [8]. Thus, early diagnosis is important to impact on natural history through the innovative disease modifying therapies.

Unfortunately, in non-endemic area, diagnosis can be delayed by 3–4 years [2] since a correct diagnosis is challenging for clinician. Patients with ATTRv amyloidosis experience multiple neurological and/or cardiovascular testing and hospitalization prior to achieve the diagnosis [9]. In 32–74% of cases, patients receive misdiagnoses [10] and undergo inadequate or inappropriate treatments. Misdiagnoses are due to the lack of family history, the heterogeneous initial clinical manifestations and nerve conduction studies (NCS) that could show some demyelinating features [11] and pathological examinations (abdominal fat and sural nerve biopsy) negative for amyloid depositions [12].

Based on disease’s red flags, suspicion index of ATTRv amyloidosis was proposed to preciously recognize ATTRv and avoid diagnostic delay [13]. Suspicion index is based on the presence of a progressive polyneuropathy in addition to at least one red flag symptom suggestive of multi-systemic involvement. However, sometimes the demonstration of a progressive neuropathy requires follow-up evaluations, risking wasting time. Moreover, some red flags (e.g., cardiomyopathy or vitreous opacities) need specialist evaluations that could be often lacking during the first neurological evaluation [14].

The aim of this study was to optimize a clinical and electrophysiological score to select patients with axonal neuropathy worthy of TTR screening.

## Methods

Thirty-five ATTRv patients and 55 patients with chronic idiopathic axonal polyneuropathy (CIAP) were retrospectively analyzed by two Italian third-level neuromuscular centers (University of Naples “Federico II” and Fondazione Policlinico Universitario A. Gemelli IRCCS of Rome). All patients underwent clinical assessment, nerve conduction study and Sanger sequencing of *TTR* gene. ATTRv patients were defined as patients with axonal polyneuropathy carrying TTR pathogenic variant. CIAP patients were defined as patients with at least of 6-month history of axonal sensory–motor polyneuropathy, resulted negative for *TTR* variant and for other causes of neuropathy through appropriate investigations [15]. In particular, all CIAP patients had no family history nor signs of hereditary neuropathy (e.i. pes cavus), no metabolic (diabetes, liver, renal or thyroid dysfunction), deficiency (vitamin B12, thiamine or pyridoxine deficiency), toxic (no history of exposure to alcohol, neurotoxic agents, drugs or chemotherapy), immunological (rheumatological, paraneoplastic or celiac disease), haematological (paraproteinemic syndrome as AL amyloidosis or POEMS) and infective (HBV, HCV, HIV) causes were identified as etiology of neuropathy.

As clinical data, we collected gender, age of onset, disease duration (time between age of onset and first evaluation) and the presence at first evaluation of (1) family history of polyneuropathy, (2) progressive disturbance in the last 6 months as perceived by patients, (3) muscle weakness, (4) positive and negative sensory symptoms (i.e., tingling and numbness), (5) autonomic symptoms (i.e., erectile dysfunction, diarrhea/constipation, nausea and vomiting, sweating abnormalities) (6) carpal tunnel syndrome (CTS) history. Moreover, we collected data about the walking impairment (0 = no walking difficulties; 1 = walking difficulties but independent; 2 = needing support; 3 = wheelchair bound).

As electrophysiological features, we collected amplitude of compound muscular action potential (CMAP; mV), distal motor latency (DML; ms) and motor nerve conduction velocity (MNCV; m/s) of the median, ulnar, tibial and peroneal nerves. Moreover, we collected amplitude of sensory action potential (SAP; μV) and sensory nerve conduction velocity (SNCV; m/s) of median, ulnar, peroneal superficial and sural nerves. Moreover, SAP and CMAP amplitude values were categorized in normal (0), reduced (1) and absent (2) according to the normal value of each center.

### Statistical analysis

**Fig. 1:**
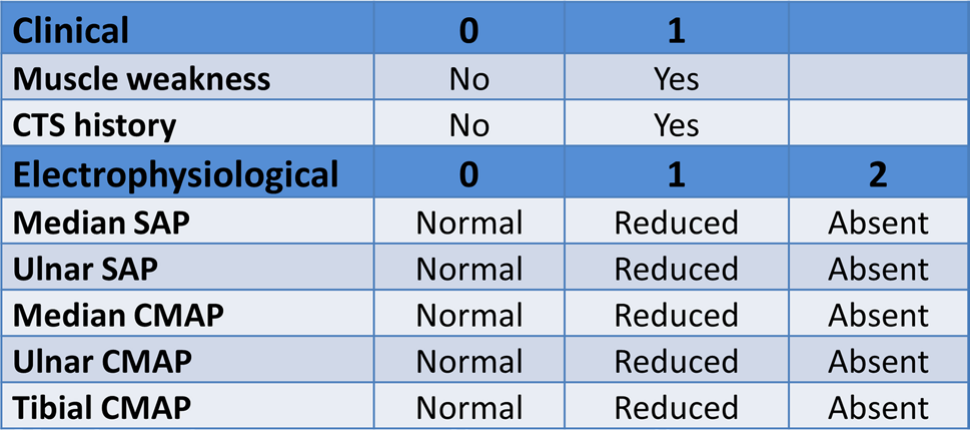
Composite clinical and electrophysiological score. *CTS* carpal tunnel syndrome, *SAP* sensory action potential, *CMAP* compound motor action potential

Descriptive statistics were based on mean ± standard deviation in the case of continuous variables and on frequencies (percentage) in the case of categorical variables. Statistical differences between ATTRv and CIAP groups were performed through Pearson's chi-squared test for categorical variables and Student’s *T* test for continuous variables. *P* values less than 0.05 were deemed as statistically significant. Based on the significant difference between two groups, a compound (clinical and electrophysiological) score (Fig. 1) was arranged (ranging from 0 to 12) assigning the highest scores to each variable that were more frequently abnormal in ATTRv patients as shown by the comparison analysis between the two groups. The score was constituted by 7 total items: motor symptoms (0 = none, 1 = present), CTS history (0–1), Median SAP (0 = normal, 1 = reduced; 2 = absent), Ulnar SAP (0–2), Median CMAP (0–2), Ulnar CMAP (0–2) and Tibial CMAP (0–2). The receiving operating characteristics (ROC) analyses were used to discriminate groups using the total score. To test the difference between ATTRv and CIAP patients with short disease duration, we performed a sub-analysis on the patients with disease duration ≤ 2 years through Student T test. All analyses were performed using STATA statistical software, version 13.

## Results

Clinical and electrophysiological findings were summarized in Table 1.

**Table 1:** Clinical and electrophysiological findings

		ATTRv (*N* = 35)	CIAP (*N* = 55)	*p value*
Clinical findings				
TTR gene mutation	V30M P64L V122I	42,8% 51.4% 5.7%	-	-
Gender	Male Female	91.4% 8.6%	83.6% 16.4%	*p* = 0.289
Family history of neuropathy	No Yes	100% 0%	100% 0%	*p* = 0.569
Progressive neuropathy	No Yes	51.4% 48.6%	47.3% 52.7%	*p* = 0.701
Walking impairment	0 1 2 3	0% 48.6% 34.3% 17.1%	0% 69.1% 25.5% 5.4%	*p* = 0.083
Muscle weakness	No Yes	14.3% 85.7%	45.5% 54.5%	***p* = 0.002**
Sensory symptoms	No Yes	2.8% 97.2%	9.1% 90.9%	*p* = 0.248
Carpal tunnel syndrome history	No Yes	42.9% 57.1%	76.4% 23.6%	***p* = 0.001**
Autonomic symptoms	No Yes	68.6% 31.4%	72.7% 27.3%	*p* = 0.672
Age of onset (years)		64.3 ± 9.9	58.2 ± 11.2	***p* = 0.011**
Disease duration (years)		4.3 ± 4.1	3.8 ± 2.7	*p* = 0.534
Electrophysiological findings				
SAP median	Normal Reduced Absent	0% 25% 75%	40.8% 34.7% 24.5%	***p* < 0.001**
SAP ulnar	Normal Reduced Absent	0% 45.5% 54.5%	31.6% 42.1% 26.3%	***p* < 0.001**
SAP sural	Normal Reduced Absent	5.9% 26.5% 67.6%	6.4% 34% 59.6%	*p* = 0.671
SAP superficial	Normal Reduced Absent	5.6% 0% 94.4%	13.9% 13.9% 72.2%	*p* = 0.139
CMAP median	Normal Reduced Absent	14.3% 60.7% 25%	73.3% 24.5% 2.2%	***p* < 0.001**
CMAP ulnar	Normal Reduced Absent	27.6% 68.9% 3.5%	73.5% 24.5% 2%	***p* < 0.001**
CMAP tibial	Normal Reduced Absent	35.3% 17.6% 47.1%	20.8% 50% 29.2%	***p* = 0.011**
CMAP peroneal	Normal Reduced Absent	17.9% 39.3% 42.8%	29.2% 35.4% 35.4%	*p* = 0.540

*p* values marked with bold indicate a statistically significant difference between the groups

*SAP* sensory action potential, *CMAP* compound motor action potential

Clinical data analysis showed that ATTRv group had more frequently motor symptoms (*p* = *0.002*) and CTS history (*p* = *0.001*) respect CIAP patients. Moreover, patients carrying a TTR variant had a later age of onset respect patients with idiopathic neuropathy (64.3 ± 9.9 *vs* 58.2 ± 11.2; *p* = *0.011*). Conversely, no other clinical differences were found between two groups (gender, disease duration, progressive disease, sensory and autonomic symptoms, walking impairment) (Table 1).

**Fig. 2:**
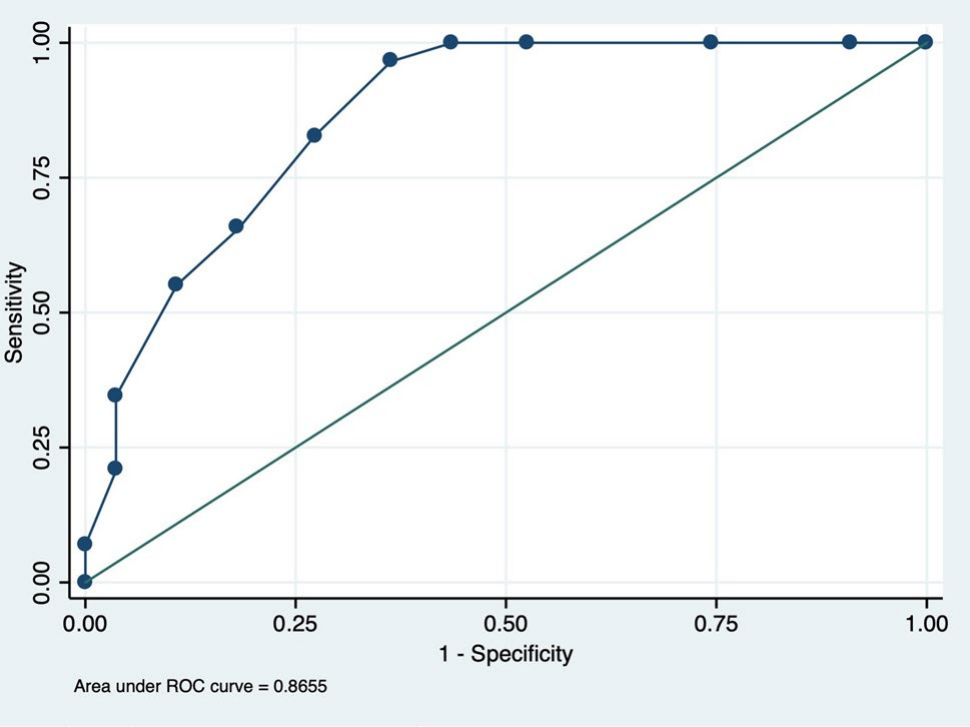
ROC analysis of composite score. ROC analysis of composite score in patients with ATTRv and CIAP patients showing an area under the curve (AUC) of 0.8655

Electrophysiological findings analysis showed that ATTRv patients had a more reduced amplitude of SAP and CMAP in all examined nerves (*p* < *0.05*) (Table 2). In detail, ATTRv group presented more frequently absent CMAP in tibial nerve (47% *vs* 29%), SAP in median (75% *vs* 24%) and ulnar (54% *vs* 26%) nerves, and more frequently reduced CMAP in median (75% *vs* 26%) and ulnar (82% *vs* 26%) CMAP (Table 1). Moreover, significant differences between two groups were MNCV across the elbow in the ulnar nerve and DML of peroneal nerve (*p* < *0.05*) (Table 2). Using ROC analysis, we established that the total score that best separated ATTRv patients from CIAP was a value ≥ 5 (AUC = 0.86, Fig. 2) with a sensitivity of 96.6% and a specificity of 63.6%. In particular, in our cohort, a total score ≥ 5 points was present in 96.6% ATTRv patients and in 36.4% CIAP patients. Lastly, the difference between the two groups with disease duration ≤ 2 years showed that the ATTRv patients had a greater score (11 patients; 7.4 + 1.2) respect to the CIAP patients (24 patients; 4 + 3.1) (*p* < 0.001).

**Table 2:** Detailed electrophysiological findings

		ATTRv	CIAP	*p* value
Median nerve	SAP (μV)	3.2 ± 2	17 ± 13.3	***p* < 0.001**
	SNCV (m/s)	44.2 ± 8.2	43.6 ± 7.5	*p* = 0.852
	DML (ms)	5 ± 1.3	4.6 ± 1.5	*p* = 0.340
	dCMAP (mV)	2.9 ± 2.6	8.6 ± 3.4	***p* < 0.001**
	pCMAP (mV)	2.6 ± 2.4	7.9 ± 3.4	***p* < 0.001**
	MNCV (m/s)	44.2 ± 6.3	45.1 ± 8.4	*p* = 0.108
Ulnar nerve	SAP (μV)	4 ± 2.7	15.4 ± 12.3	***p* < 0.001**
	SNCV (m/s)	47.6 ± 5.8	46 ± 7.6	*p* = 0.544
	DML (ms)	3.3 ± 0.8	3.2 ± 0.8	*p* = 0.683
	dCMAP (mV)	5.4 ± 3.7	9.5 ± 3.8	***p* < 0.001**
	p1CMAP (mV)	5.1 ± 3.2	8.7 ± 3.6	***p* < 0.001**
	p2CMAP (mV)	5 ± 2.9	8.1 ± 3.5	***p* = 0.001**
	MNCV1 (m/s)	49.8 ± 8	51.8 ± 7.7	*p* = 0.304
	MNCV2 (m/s)	39.7 ± 9.2	43.9 ± 7	***p* = 0.042**
Tibial nerve	DML (ms)	5 ± 1.5	5.6 ± 1.5	*p* = 0.165
	dCMAP (mV)	2.3 ± 3	5.2 ± 5.2	***p* = 0.022**
	pCMAP (mV)	1 ± 2.6	3.9 ± 4.4	*p* = 0.291
	MNCV (m/s)	37.9 ± 3.9	36.7 ± 5.9	*p* = 0.621
Peroneal nerve	DML (ms)	3.8 ± 1	5.0 ± 1.9	***p* = 0.024**
	dCMAP (mV)	2.0 ± 1.9	4.4 ± 3.8	***p* = 0.006**
	pCMAP (mV)	1.9 ± 2.1	3.8 ± 3.3	***p* = 0.026**
	MNCV (m/s)	39.6 ± 11.7	38.7 ± 7.2	*p* = 0.747
Sural nerve	SAP (μV)	3.2 ± 1.8	3.9 ± 2.8	*p* = 0.460
	SNCV (m/s)	45.4 ± 3.3	46.6 ± 7.1	*p* = 0.523

*p* values marked with bold indicate a statistically significant difference between the groups

*SAP* sensory action potential, *SNCV* sensory nerve conduction velocity, *DML* distal motor latency, *(d/p)CMAP*(distal/proximal) compound motor action potential, *MNCV* motor nerve conduction velocity

## Discussion

The neuropathy in ATTRv patients represents one of the most disabling and progressive conditions and sometimes electrophysiological findings can misinterpreted by clinician although the neuropathy is due to a primary axonal degeneration [11]. Our study aimed to mark peculiar clinical and electrophysiological characteristics which can help clinicians to suspect ATTRv among patients with axonal polyneuropathy.

Clinical findings showed that ATTRv patients referred more frequently motor symptoms (86% *vs* 54%) and CTS history (57% *vs* 24%) respect patients with CIAP. These results confirmed that ATTRv patients have a precocious involvement of motor system respect CIAP patients which complain especially sensory symptoms [16]. In fact, although statistical analysis missed to reach significance, only 50% (*vs* 70%) of our ATTRv patients can walk independently.

Conversely, in our cohort, autonomic symptoms did not represent a discriminative feature. In fact, our population was constituted by late-onset ATTRv patients [17] in which autonomic involvement at the disease onset is often subtle and undetected if not adequately investigated and become clinically prominent in advanced stage [1]. Another possible explanation for this lacking significant was that the autonomic dysfunction was detect through the reported symptoms during patient’s interview and not by appropriate questionnaire or specific instrumental test (e.g., tilt test, skin sympathetic response).

Lastly, disease progressivity unexpectedly did not differ between ATTRv and CIAP patients. The reason of this result could be due to the retrospectively design of the study. In fact, the disease progression was considered as perceived by patients at first evaluation and not by follow-up evaluations. As occurs in other conditions, in which the patient perception of health status does not parallel functional and disability measures [18], CIAP patients could perceive their neuropathy as progressive disease as well.

Electrophysiological findings showed that ATTRv patients, although they had the same disease duration of CIAP patients, had a greater reduction of amplitude of potentials in all nerves with a more frequently absence of potential at lower limbs and reduction at upper limbs. Our data confirm that axonal degeneration is the primary patho-mechanism in ATTRv disease and suggests early involvement of upper limbs nerves respect to CIAP patients in which simultaneous development of upper and lower extremity rarely occurs [19]. Although ATTRv neuropathy is defined as length-dependent, the early involvement of upper limb nerves could be the expression of a ganglionopathic pattern damage [20]. In ATTRv amyloid, accumulation starts in dorsal root ganglia and nerve roots and afterward amyloid deposits spread through a proximo-distal gradient over time [11].

Moreover, our study confirmed the role of CTS history as red flag of ATTRv as it could precede by several years the onset of polyneuropathy [21]. Of interest, our electrophysiological data findings in symptomatic ATTRv patients did not show a greater SNCV slowing and DML prolongation in median nerve respect to CIAP patients as expected. Our findings suggest that in a patient with polyneuropathy, the clinical history of CTS is important in the suspicion of ATTR rather than electrophysiological findings of CTS. In fact, the CTS physiopathology in ATTRv patient seems to have a peculiar behavior respect the idiopathic CTS. The ultrasound results showed that CTS in ATTRv is characterized by a peculiar mismatch between electrophysiological and ultrasound abnormalities of the median nerve at wrist, differentiating from idiopathic CTS, in which ultrasound findings mirror electrophysiology severity [22]. Altogether, we can suppose that the entrapment injury of the median nerve can occur in pre-symptomatic stage through the deposition of amyloid in the carpal ligament [23], but contextually, there is already a systemic damage of nerves that starts proximally [24].

Based on this peculiar characteristic of ATTRv patients, we arranged a compound clinical and electrophysiological score. A total score ≥ 5 allows to identify with a sensitivity over 95% ATTRv patients among subject with chronic axonal polyneuropathy. We have decided to set a cut-off with higher sensitivity respect the specificity, since the score was arranged as a screening tool. We opted to have more false positive respect to lose the possibility to detect an ATTRv patient, given that the disease is debilitating but curable especially in the early stage [25]. Moreover, the score is able to discriminate ATTRv also in patients with short disease duration (≤ 2 years), strengthening that the ATTRv leads to a severe neuropathy since the first year of disease.

The score emphasizes the predominant motor involvement in ATTRv disease, respective to CIAP, and this difference might cause an imbalance between the two populations. However, in front of a single patient with neuropathy, the severity of motor involvement can be difficult to valorize. In fact, CIAP still represents a common misdiagnosis for ATTRv patients [26]. Our study identified the principal differences between the two groups and valorized them in a compound score which can help clinician through a specific cut-off to recognize patients deserving TTR genetic analysis. Moreover, the score is easy to perform during clinical and/or electrophysiological examination and it does not require other special exam (e.g., cardiac imaging or ophthalmological examination to detect cardiomyopathy and vitreous corpus respectively).

If the score can influence marginally the choice to perform genetic analysis in a third-level center, where TTR genetic test is easily accessible, conversely it can help physician in primary centers, where the patients are evaluated for the first time and genetic test can be difficult to perform. Especially in this context, the application of the compound score in patients with sensory–motor neuropathy may have a major role, representing a first screening tool to drive the choice of referring patients in an amyloidosis center, avoiding wasting time and therefore shortening the time to reach a correct diagnosis.

In conclusion, although the small number of patients and the needing to confirm the result through a prospective study, our study suggests that some clinical and electrophysiological features in sensory–motor neuropathy patients, such as CTS history, relevant motor impairment and a greater axonal loss with a precocious upper limb involvement, should alert the clinicians [27]. Applying our score to patients with sensory–motor neuropathy, clinicians could easily establish if patients deserve *TTR* genetic analysis. This score can be easily performed also during electrophysiological evaluation and can be extremely useful in those area where genetic analysis is not easily accessible.
